# Elucidation of the Interaction Mechanism with Liposomes of gH625-Peptide Functionalized Dendrimers

**DOI:** 10.1371/journal.pone.0112128

**Published:** 2014-11-25

**Authors:** Annarita Falanga, Rossella Tarallo, Thomas Carberry, Massimiliano Galdiero, Marcus Weck, Stefania Galdiero

**Affiliations:** 1 Department of Pharmacy & CIRPEB & DFM Scarl, University of Naples “Federico II”, Naples, Italy; 2 Molecular Design Institute and Department of Chemistry, New York University, New York, New York, United States of America; 3 Department of Experimental Medicine, Second University of Naples, Naples, Italy; Universidad de Castilla-La Mancha, Spain

## Abstract

We have demonstrated that amide-based dendrimers functionalized with the membrane-interacting peptide gH625 derived from the herpes simplex virus type 1 (HSV-1) envelope glycoprotein H enter cells mainly through a non-active translocation mechanism. Herein, we investigate the interaction between the peptide-functionalized dendrimer and liposomes composed of PC/Chol using fluorescence spectroscopy, isothermal titration calorimetry, and surface plasmon resonance to get insights into the mechanism of internalization. The affinity for the membrane bilayer is very high and the interaction between the peptide-dendrimer and liposomes took place without evidence of pore formation. These results suggest that the presented peptidodendrimeric scaffold may be a promising material for efficient drug delivery.

## Introduction

The cellular membrane plays an essential role in controlling the transport of external molecules into the cell interior. It acts as a selective barrier that only allows the passage of selected molecules. Non-specific and non-disruptive penetration across the membrane without protein mediation is typically achievable only by small molecules, while larger ones generally induce significant disruption of the lipid bilayer structure [1]. Thus, there is considerable interest to pave the way to strategies able to translocate synthetic molecules into and through the membrane for drug delivery, biosensing, and other biomedical applications, without permanently disrupting the membrane and the risk of inducing cell death [2]. The success of novel strategies for health care therapies relies on the development of delivery vehicles capable of improving the therapeutic index of biologically active molecules as well as diagnosing at the disease site of interest. At the present, most active compounds are not deemed therapeutically effective due to their inability to reach the target tissue. A variety of drug delivery carriers including polymer microcapsules, liposomes, polymer conjugates, and nanoparticles are in pre-clinical and clinical development to improve many drug bioavailability, especially for cancer treatment [3], [4]. Most drug carriers lack the potential for orthogonal multi-functionalization, a prerequisite for theranostics and are often not well-defined compounds but polydisperse mixtures, rendering the analysis of structure-property relationships challenging. These drawbacks limit their potential use in theranostics. One class of biomaterials that can serve as drug carrier or, more generally as a platform for theranostics, and that has the potential to overcome these limitations is dendrimers.

Dendrimers are well-defined synthetic hyperbranched macromolecules which possess a high number of active termini that defines their properties and functions [5]. As a result of perfect branching, dendrimers have the highest number of terminal functionalities of any polymeric material at a given molecular weight and are monodisperse. Comparing the features of dendrimers with those of linear polymers, it was shown that the dendrimer architecture presents several advantages for drug delivery [6], [7]: (i) low polydispersity allowing for reproducible pharmacokinetic behavior; ii) globular shape of higher generation dendrimers affecting their biological and rheological properties; and iii) controlled multivalency which can be used to attach several molecules, e.g. drugs, imaging agents, cell-penetrating peptides, targeting groups, and/or solubilizing moieties. The possibility to introduce several functionalities into the dendrimer structures has opened the door for their applications in theranostics [8]. Over the past decade, the relationships between dendrimer architecture, biocompatibility, circulation time, and release kinetics have been partially elucidated allowing the development of general principles for the design of dendrimers as “ideal” drug delivery carriers. These include PEGylation to increase water solubility, permeability, stability and dendrimer size (leading to tunable retention and biodistribution characteristics), the internalization of therapeutic agents into the void space between the branches or the covalent attachment of them to surface groups, and the addition of targeting moieties bound to the dendrimer surface can be used to preferentially treat disease cells with specific over-expressed receptor targets.

Dendrimers usually cross cell barriers by endocytosis [9], [10], thus they are entrapped in endososomes and only a small amount of the active drug is able to reach the intracellular target. We recently reported that virally derived peptides could significantly help in solving this problem as many viruses have evolved rather efficient systems for endosomal release. Viruses can enter cells either through an endosomal pathway or via direct fusion of the plasma membrane through the activity of membranotropic peptides [11], [12]. Great attention has been devoted to the study of hydrophobic peptides that efficiently traverse biological membranes, promoting lipid-membrane reorganizing processes, such as fusion or pore formation, and involving temporary membrane destabilization and subsequent reorganization [13], [14], [15], [16], [17], [18], [19], [20]. These peptides are significantly different from the well-known cell penetrating cationic peptides such as TAT, which do not translocate spontaneously across bilayers but rather are taken up by cells via endocytosis [21]. According to our previously reported data [22], [23], coupling of a poly(amide)-based dendrimer [24], [25] to viral peptide gH625 enabled the non-specifically crossing of the membrane bilayer through an energy-independent process, without showing evidence of endocytosis, poration, or cytotoxicity. Translocation mediated by gH625 may be very useful in the delivery of drugs; the mechanism of spontaneous translocation is not yet well understood.

The translocation of gH625 dendrimers through lipid bilayers could provoke changes in the bilayer which need to be taken into account in the design of a drug controlled release delivery system. To understand the mechanism of our HSV-1 derived peptide-dendrimer to mediate the cell membrane crossing, it is necessary to understand its interactions with lipid bilayers [26]. Liposomes are excellent model systems for biological experiments because of their simple and membrane-like composition, easy preparation, biodegradability, biocompatibility and acceptable stability over time. By examining the interactions between liposomes and dendrimers, conclusions can be drawn about biological processes such as membrane fusion and translocation and ultimately transport pathways for drug delivery. In the literature, several modes of interactions between dendrimers and liposomes are described [27], [28], [29], [30], [31], [32], [33], [34], [35], [36], [37], [38]. Dendrimers can either pass through the lipid bilayer or dendrimer-lipid micelles are created [39]. Some dendrimers can interact with lipids by hydrophobic interactions between lipid acyl chains and the hydrophobic dendrimer interior [40], [41]. It was shown that the strength of the interaction mainly depends on the size and charge of the molecule [42], [43], [44] and the phase of lipids [45].

This contribution describes the effect of the gH625 dendrimer on lipid bilayers composed of cholesterol (Chol) and neutral phospholipids such as phosphatidylcholine (PC). Surface plasmon resonance (SPR), fluorescence spectroscopy, and isothermal titration calorimetry (ITC) were used to elucidate the dendrimer/membrane interactions in order to push further the design of new drug delivery systems that consist of dendrimers incorporating bioactive molecules. These studies are of interest in understanding the interaction of gH625-dendrimer with native cell membranes, and complement previous studies showing that the gH625-dendrimer is able to pass through and does not disrupt the biological membrane.

## Materials and Methods

### Materials

Fmoc-protected amino acids, coupling reagents, and Rink- amide *p*-methylbenzhydrylamine (MBHA) resin were purchased from Calbiochem-Novabiochem (San Diego, CA, USA). Fmoc-l-propargylglycine (Fmoc-PrA-OH) was purchased from NeoSystem (Tysons Corner, VA, USA). The phospholipid phosphatidylcholine (PC), 1-hexadecanoyl-2-(6,7-dibromooctadecanoyl)-sn-glycero-3-phosphocholine (6,7 Br-PC), 1-hexadecanoyl-2-(9,10-dibromooctadecanoyl)-sn-glycero-3-phosphocholine (9,10 Br-PC), 1-hexadecanoyl-2-(11,12-dibromooctadecanoyl)-sn-glycero-3-phosphocholine (11,12 Br-PC) were purchased from Avanti Polar Lipids (Birmingham, AL, USA). Cholesterol, Triton-X100 and 3-propyl-2-(5-(3-propyl)-2(3H)-benzothiazolidene-1,3-pentadienyl), iodide (diS-C_3_-5) were obtained from Sigma (St. Louis, MO, USA). All other chemicals were purchased from Sigma-Aldrich (St Louis, MO, USA), Alfa Aesar (Ward Hill, MA, USA), or TCI International (Portland, Oregon, USA). Ultrafiltration membranes were purchased from Millipore. Analytical and semi-prep reverse-phase high-performance liquid chromatography (HPLC) was performed on a Shimadzu LC8 pump setup using Phenomenex C4 (4.6×150 mm-5 µm, 10×250 mm-10 µm) columns.

### gH625-dendrimer preparation

The dendrimer and the peptide have been synthesized as previously reported [22], [23]. To obtain the peptidodendrimer, a 1∶1 methanol/water solution of peptide gH625–PrA (660 µl, 36 equiv), an aqueous solution of CuSO_4_·5H_2_O (10 µl, 1.46 mM, 1 equiv), and an aqueous solution of sodium ascorbate (50 µl, 1.17 mM, 4 equiv) were added to the dendrimer (50 µg, 0.0146 µmol) in a 1∶1 water/methanol solution (280 µl). The mixture was left stirring for one hour at 40°C and for two days at RT. The compound was purified twice by ultrafiltration in water:methanol:DMSO 50∶48∶2 versus 30,000 molecular weight cut-off membranes and then by reverse phase HPLC on a C4 column with water (0.1% TFA) and acetonitrile (0.1% TFA) from 30 to 95% over 20 min at 5 mL/min. IR (cast on poly(ethylene)): 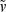 = 3295, 1658, 1545, 1471, 1203 cm^−1^. No peak at 2098 cm^−1^ was observed. The functionalization of the gH625-dendrimer was confirmed by determining the amount of peptide attached via UV analysis (ε_gH625_ = 7000 M^−1^cm^−1^ at λ = 280 nm) and comparing the result to the amount of dendrimer and peptide initially used for the reaction (18 mol peptide per mol dendrimer). The functionalization of the peptidodendrimer, and thus the reaction between the azide and the alkyne, was also confirmed by following the ^1^H NMR (MeOD, 600 MHz) chemical shift of the triplet representing the CH_2_N_3_ protons of the dendrimer from 3.37 to 5.34 ppm after reaction.

### Liposome preparation

Phospholipids and fluorescent probes were dissolved in organic solvents. Then the solvent was evaporated to obtain a lipid film which was hydrated with an appropriate volume of 10 mM HEPES buffer at pH 7.4. Small unilamellar vesicles (SUVs) was prepared from dry lipid films were suspended in buffer by vortexing for 1 h, then the lipid suspension was sonicated for 30 minutes. Lipid concentrations of liposome suspensions were determined by phosphate analysis as previously reported [16]. The final lipid concentration was 1 mM.

### Tryptophan fluorescence measurements

Tryptophan fluorescence increases with an increase of the environment hydrophobicity and a blue shift of the emission maxima was observed. Emission spectra of the gH625 peptide and of the gH625-dendrimer (4 µM in peptide), containing the tryptophan residue, in the absence or presence of target vesicles (PC/Chol  = 55/45) were recorded between 300 and 400 nm with an excitation wavelength of 295 nm.

The degree of association with the lipid vesicles was determined by adding lipid vesicles to 4 µM of the desired compounds. Fluorescence intensity was measured as a function of the lipid/peptide molar ratio, in three to four separate experiments. The fluorescence values were corrected by taking into account the dilution factor corresponding to the addition of microliter amounts of liposomes and by subtracting the corresponding blank. The lipid/peptide molar ratio was 200∶1.

The binding of gH625 and gH625-dendrimer to membranes can be described as a partition equilibrium: X_b_ = K_p_C_f_ where K_p_ is the apparent partition coefficient in units of M^−1^, X_b_ is the molar ratio of bound molecules per total lipid and C_f_ is the equilibrium concentration of the free molecules in solution. In order to calculate X_b_, we estimated F_∞_, the fluorescence signal obtained when all the molecules are lipid-bound, either from the plateau region of the titration curve or from a double reciprocal plot of F (total molecule fluorescence) versus C_L_ (total concentration of lipids), as previously suggested by Schwarz et al. [46]. (F_∞_ was obtained by extrapolation of a double reciprocal plot of the total molecule fluorescence vs. the total lipid concentration in the outer leaflet, i.e. 1/F vs. 1/0.6C_L_). Knowing the fluorescence intensities of the free and bound forms of the molecules of interest, the fraction of membrane-bound molecules, f_b_, can be determined from f_b_ =  (F-F_0_)/(F_∞_-F_0_), where F represents the fluorescence of molecules after the addition of the vesicles and F_0_ represents the fluorescence of the unbound molecules. Determining f_b_ allows us to calculate the equilibrium concentration of free molecules in solution, C_f_, as well as the extent of molecules binding X_b_. It was assumed that the molecules were initially partitioned only over the outer leaflet of the SUV (60% of the total lipid). Therefore, values of X_b_ were corrected as follows: X_b_^*^  =  X_b_/0.6.

**Figure 1:**
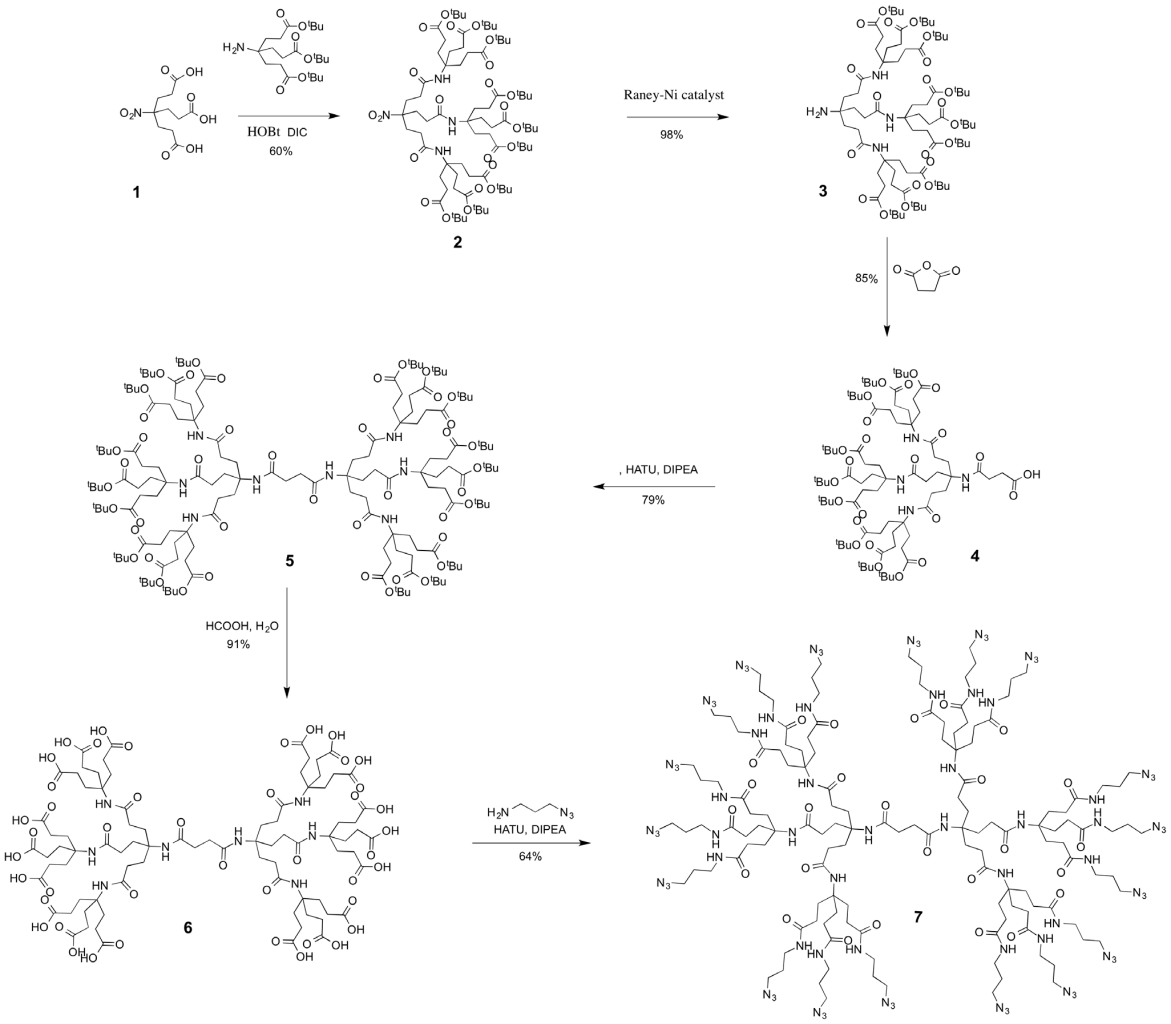
Scheme of the octadecaazide dendrimer synthesis.

The curve resulting from plotting X_b_^*^ versus the concentration of the free molecules, C_f_, is referred to as the conventional binding isotherm. Plots of X_b_^*^ versus C_f_ yield straight lines with the slope corresponding to K_p_ if a simple partition equilibrium is observed. A deviation is expected for the binding of molecules that self-associate at the membrane surface corresponding to binding isotherms that are not straight lines but deviate to increased binding at higher molecule concentrations. If enough data points of C_f_ can be collected at very low free molecule concentrations, the surface partition coefficients, K_p_, can be estimated from the initial slopes of the curves (Figure 1).

### Tryptophan quenching by acrylamide

Aliquots of a 4 M solution of the water-soluble quencher acrilamide were added to the solution containing gH625 or gH625-dendrimer (4 µM in peptide) in the absence or presence of liposomes (PC:Chol 55∶45, 8•10^−4^ M) at a lipid/peptide molar ratio of 200: 1. The maximal concentration of acrylamide is 0.2 mM. Fluorescence was measured at an excitation wavelength of 295 nm to reduce acrylamide absorbance (and the resulting inner filter effect), and emission at a wavelength of 340 nm to eliminate interference from the Raman band of water. The data were analyzed according to the Stern-Volmer equation [47], F_0_/F  = 1 + K_sv_ [Q], where F_0_ and F represent the fluorescence intensities in the absence and the presence of the quencher (Q), respectively, and K_sv_ is the Stern-Volmer quenching constant, which is a measure of the accessibility of tryptophan to acrylamide. Considering that acrylamide does not significantly partition into the membrane bilayer, the value for K_sv_ can be considered to be a reliable reflection of the bimolecular rate constant for collisional quenching of the tryptophan residue present in the aqueous phase. Accordingly, K_sv_ is determined by the amount of non-vesicle-associated free molecule as well as the fraction of the molecule residing on the surface of the bilayer.

### Tryptophan quenching experiments with Br-PC

Tryptophan is sensitive to its environment and has been previously utilized to evaluate peptide localization in the membrane [48]. Emission spectra of the gH625-dendrimer in the absence or presence of target vesicles (PC/Chol  = 55/45) were recorded between 300 and 400 nm with an excitation wavelength of 295 nm. Br-PC employed as quencher of tryptophan fluorescence is suitable for probing membrane insertion of peptides, since it acts over a short distance and does not drastically perturb the membrane [20]. The peptidodendrimer was added (final concentration of 222 nM) to 2 mL of SUVs, thus establishing a lipid:peptide molar ratio of 100∶1. After two minutes of incubation at room temperature, an emission spectrum of the tryptophan was recorded with the excitation set at 295 nm. SUVs composed of PC/Chol that contained 25% of either 6,7 Br-PC, or 9,10 Br-PC or 11,12 Br-PC were used. Three separate experiments were conducted. In control experiments, the peptidodendrimer in PC/Chol (55/45) SUVs without Br-PC were used.

### Membrane permeability studies

Membrane destabilization, in the form of diffusion potential collapse, was detected fluorimetrically as described previously [49]. Briefly, a liposome suspension (SUV = PC:Chol 55∶45, 0.1 mM), prepared in “K^+^+buffer”, (50 mM K_2_SO_4_, 25 mM HEPES-SO_4_ pH 6.8) was added to an isotonic buffer, K^+^-free buffer (50 mM Na_2_SO_4_, 10 mM HEPES-SO_4_ pH 6.8), to which the dye diS-C_3_-5 (final concentration 1×10^−6^ M) was added. Subsequent addition of valinomycin (final concentration 1×10^−7^ M) creates a negative diffusion potential inside the vesicles by a selective efflux of K^+^ ions, resulting in a quenching of the dye's fluorescence. The addition of a permeable compound induces membrane permeability toward all the ions in solution causing dissipation of the diffusion potential, monitored by an increase of fluorescence. Compound concentrations varied from 5 10^−6^ M to 7 10^−5^ M. Fluorescence was monitored using excitation at 620 nm and emission at 670 nm. The percentage of fluorescence recovery (F_t_) was defined as shown in Equation 1.



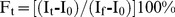
(Eq. (1))


Ι_t_ is the fluorescence observed after addition of the dendrimer-peptide at time t, Ι_0_ is the fluorescence after addition of valinomycin, and Ι_f_ is the total fluorescence prior to addition of valinomycin. The peptide melittin was used as a positive control in this experiment.

### Binding analysis by surface plasmon resonance (SPR)

SPR experiments were carried out with a BIAcore 3000 analytical system (Biacore, Uppsala, Sweden) using the HPA sensor chip. The HPA sensor chip contains hydrophobic alkanethiol chains, which are covalently bound to its gold surface, and a lipid heteromonolayer created by introducing liposomes to the chip. The complete coverage of the surface with a polar lipid monolayer generates a membrane-like environment where analytes in aqueous buffer interact with a lipid monolayer. The experimental protocol used, was previously described by Mozsolits *et al*. [50]. The running buffer used for all experiments was 5 mM HEPES 100 mM NaCl (pH 7.4); the washing solution was 40 mM N-octyl *β*-D-glucopyranoside. All solutions were freshly prepared, degassed, and filtered through 0.22 µm pores. The operating temperature was 25°C. After cleaning as indicated by the manufacturers, the BIAcore 3000 instrument was left running overnight using Milli-Q water as eluent to thoroughly wash all liquid-handling parts of the instrument. The HPA chip was then installed, and the alkanethiol surface was cleaned by an injection of the nonionic detergent N-octyl *β*-D-glucopyranoside (25 µl, 40 mM) at a flow rate of 5 µl/min. PC/Chol (55/45 w/w) SUVs (80 µl, 0.5 mM) were then applied to the chip surface at a flow rate of 2 µl/min. To remove any multilamellar structures from the lipid surface, we used NaOH 10 mM and increased the flow rate to 50 µl/min, which resulted in a stable baseline corresponding to the lipid monolayer linked to the chip surface. The negative control albumin bovine serum (BSA) was injected (25 µl, 0.1 mg/µl in PBS) to confirm complete coverage of the nonspecific binding sites. gH625-dendrimer solutions at the concentration of 0.07, 0.11, 0.22, 0.28, 0.33, 0.44, 0.55 nM (75 µl at a flow rate of 30 µl/min) were injected onto the lipid surface. HEPES alone then replaced the peptide solution for 15 min to allow gH625-dendrimer dissociation. SPR detects changes in the reflective index of the surface layer of peptidodendrimer and lipids in contact with the sensor chip. A sensorgram is obtained by plotting the SPR angle against time. This change in the angle is then translated to response units. Analysis of the peptidodendrimer lipid binding event was performed from a series of sensorgrams collected at different peptide concentrations.

The sensorgrams for each gH625-dendrimer lipid interaction were analyzed by curve fitting using numerical integration analysis. The BIA evaluation was used to perform complete kinetic analyses of the gH625-dendrimer sensorgrams and results were compared with those obtained for the peptide alone which were previously reported [18].

### Isothermal calorimetry

Isothermal titration calorimetry was carried out on a MicroCal-ITC 200 calorimeter. Solutions were degassed under vacuum for ten minutes with gentle stirring prior to use. The heat of dilution values were obtained in control experiments by injecting either the peptidodendrimer or the lipid solutions into buffer and the heats of dilution were substracted from the heats determined in the corresponding peptidodendrimer-lipid experiments. Two kinds of experiments were performed; in the first the calorimeter cell contains lipid vesicles and the peptidodendrimer is injected into the cell (lipid 0.033 mM and gH625-dendrimer 17 µM); while in the second, the two solutions are exchanged (gH625-dendrimer 2.8 µM was placed in the 280 µL reaction cell, while SUVs 2 mM (PC:Chol 55∶45) in 5 mM Hepes 100 mM NaCl (pH 7.4) were placed in a 40 µL syringe). In the second experiment the LUVs were added into the gH625-dendrimer solution via 20 injections of 2 µl per injection. The injections were pre-programmed at 150 sec intervals and were performed automatically at 25°C under stirring at 1000 rpm. The exotermic heat flow (dQ/dt) data were collected every second for the first 100 sec after each injection and every 5 sec for the remaining time interval and were analysed using the inbuilt Origin software.

## Results

### Synthesis of the gH625-dendrimer

The octadecaazide dendrimer was synthetized as previously reported [22] and as shown in Figure 1. Briefly, the second generation Newkome-style dendron 2 was first functionalized at the amine terminus with succinic anhydride to afford the hemisuccinate dendron 3. Coupling dendrons 2 and 3 by using 2-[7-aza-1H-benzotriazol-1-yl]-1,1,3,3-tetramethyluronium hexafluorophosphate (HATU) as coupling agent afforded the symmetrical dendrimer 4. Acidic deprotection of the terminal tert-butyl esters resulted in the formation of the octadecaacid dendrimer 5. Reaction between the terminal carboxylic acid groups and an azido-terminated amine and an azido-terminated amine linker afforded the octadecaazide dendrimer 6 (Figure 1).

**Figure 2:**
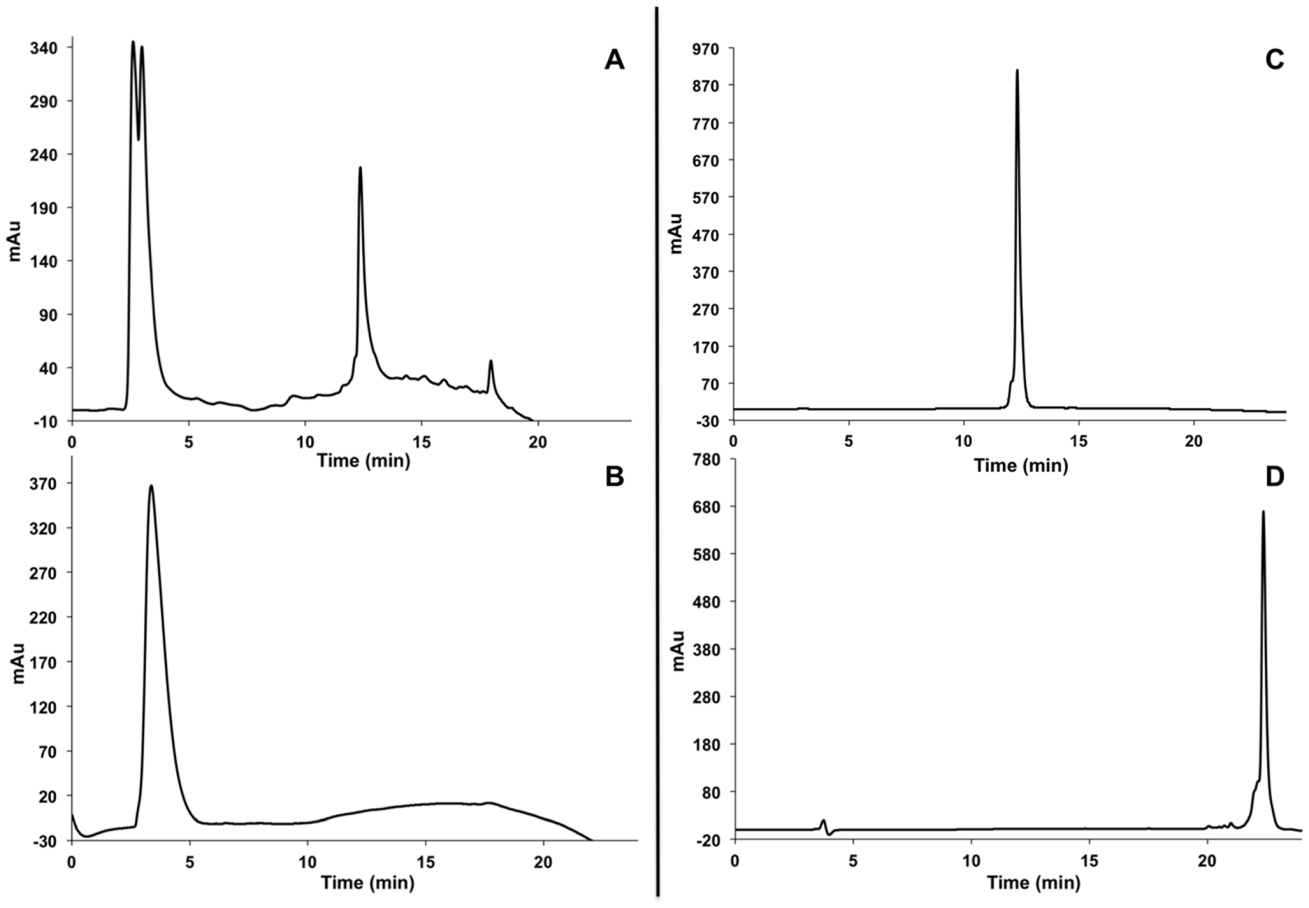
HPLC traces of gH625-Dendrimer crude (panel A), gH625-Dendrimer pure (panel B), gH625 pure (panel C) and dendrimer pure (panel D).

The gH625 peptide sequence was synthesized with a propargylglycine residue (PrA) at the C terminus to provide a handle for the copper-catalyzed azide–alkyne cycloaddition (CuAAC). Functionalization of the dendrimer was performed in a water/methanol solution (1∶1 v/v) by using CuSO_4_·5H_2_O as catalyst and sodium ascorbate as reducing agent. The obtained peptide-dendrimer was purified by HPLC as reported in the experimental section. The obtained profiles are reported in Figure 2. The amount of peptide functionalization was determined to be 71% by UV analysis (ε = 7000 M^−1^ cm^−1^ at λ = 280 nm). IR analysis showed the disappearance of the azide stretch at 2098 cm^−1^ suggesting that all azides were consumed within the instrumental error range, thus suggesting high functionalization of the dendrimer with peptides. Analysis of the NMR spectrum suggested at least 57% functionalization, corresponding to 10–11 peptides per dendrimer, however the actual value is higher due to the peak at 3.37 ppm overlapping with residual solvent and peptide backbone peaks, and possible suppression of the triazole ring due to aggregation of the dendrimers in solution. These three methods suggest that the degree of functionalization is above 70% and potentially quantitative.

### Tryptophan fluorescence measurements

**Figure 3:**
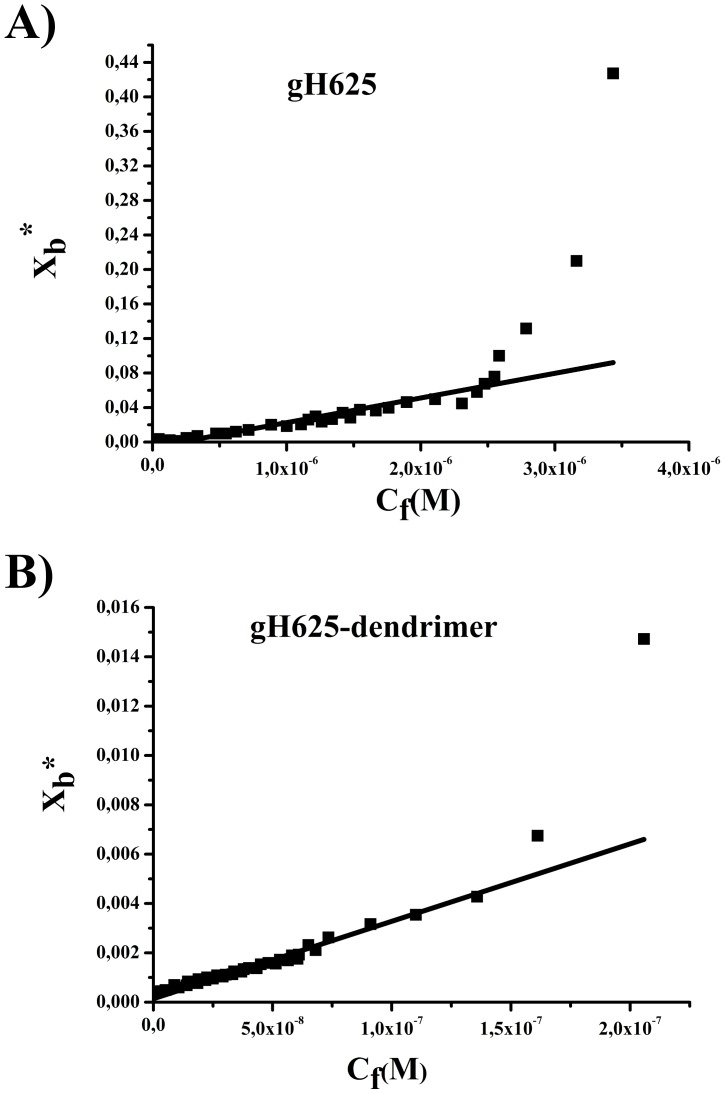
Binding isotherms obtained plotting X_b_^*^ versus C_f_ for gH625 and gH625-Dendrimer.

In order to evaluate the degree of penetration of the gH625-dendrimer into the membrane bilayer we compared the intrinsic fluorescence of gH625 alone and of gH625-dendrimer due to the presence of a tryptophan residue in the middle of the peptide sequence. For both of them, we compared the fluorescence emission spectra in the presence of PC/Chol vesicles with that in buffer (Figure 3).

It is widely accepted that the fluorescence emission of tryptophan residues increases when the amino acid penetrates a more hydrophobic environment and the maximal spectral position may be shifted toward shorter wavelengths (blue shift) [48]. For both molecules, we observed changes in the spectral properties, suggesting that the tryptophan residue of each of them is located in a less polar environment upon interaction with lipids. Emission intensity was enhanced and the maxima shifted to lower wavelengths; blue shifts of this magnitude have been observed when amphiphilic tryptophan-containing peptides interact with phospholipid bilayers and are consistent with the indole moiety becoming partially immersed in the membrane, further suggesting that the two molecules are capable of penetrating a lipid bilayer [48]. A lipid-exposed tryptophan residue located at the center of the hydrocarbon core of a bilayer exhibits a characteristic highly blue shifted emission, with a λ_max_ in the range of 315–318 nm http://pubs.acs.org/cgi-bin/article.cgi/bichaw/2003/42/i11/html/bi026697d.html - bi026697db00003#bi026697db00003. As a tryptophan residue moves toward the more polar membrane surface, λ_max_ gradually shifts to 335–340 nm. A smaller blue shift of the fluorescence for a tryptophan at the bilayer center can also be detected upon helix oligomerization inside the membrane. This shift presumably reflects the change in local environment upon replacement of contacts between tryptophan and lipid with contacts between tryptophan and polypeptide. Our data support the hypothesis that the tryptophan is located inside the membrane and moreover that oligomerization processes may have taken place.

The increase in fluorescence for tryptophan binding to membrane phospholipids was used for the binding isotherms for gH625 and the gH625-dendrimer, from which partition coefficients could be calculated. The concentrations of peptides used were low enough to cause minimal aggregation in the aqueous phase and were assumed to not disrupt the bilayer structure. SUV vesicles were used in the assay in order to minimize light scattering effects.

To determine the surface partition coefficient, the fluorescence intensities were converted to moles of bound peptide per moles of lipid and plotted as a function of the free peptide concentration as described in material and methods (Figure 3).

As partition coefficients depend on the concentration of lipid accessible to peptide, the curves obtained by plotting X_b_^*^ (the molar ratio of bound peptide per 60% of the total lipid) vs C_f_ (the equilibrium concentration of free peptide in the solution) are referred to as the conventional binding isotherms. We observed a non-linear relationship indicating accumulation of the two compounds on the surface which cannot be explained as a simple phenomenon without cooperative association. This behavior is the hallmark for peptides that self-associate at membrane surfaces upon partitioning. If aggregation occurred only in water but not in the bilayer phase, the opposite course of the isotherms should be expected: a steep rise at the origin, followed by pronounced flattening; thus, the shape of the isotherms obtained could be interpreted as reflecting a process whereby peptides first incorporate into the membrane and then aggregate there within. Moreover, there was no evidence of aggregation in water at the concentration used in this experiment. In the isotherms obtained, the total extent of incorporation (X_b_^*^) slowly increases until a critical concentration is reached, where massive internal aggregation apparently starts to develop.

The surface partition coefficients K_p_ were estimated by extrapolating the initial slopes of the curves to C_f_ values of zero; curves are shown in Figure 3.

The K_p_ values for gH625 and gH625-Dendrimer are shown in Table 1. The K_p_ value obtained for gH625 is 2.8 10^4^, indicating that the tryptophan in gH625 is embedded in the bilayer and thus that most of the peptide gH625 is located inside the bilayer. The K_p_ value for gH625-dendrimer is 3.4 10^4^, indicating that also in the functionalized dendrimer the tryptophan residue of the peptide is stably inserted inside the bilayer.

**Table 1:** Partition coefficient for the binding of gH625 and gH625-dendrimer with PC/Chol obtained by fluorescence studies.

	gH625	gH625-dendrimer
K_p_	2.8 10^4^±0.2	3.4 10^4^±0.1

### Quenching of tryptophan by acrylamide

**Figure 4:**
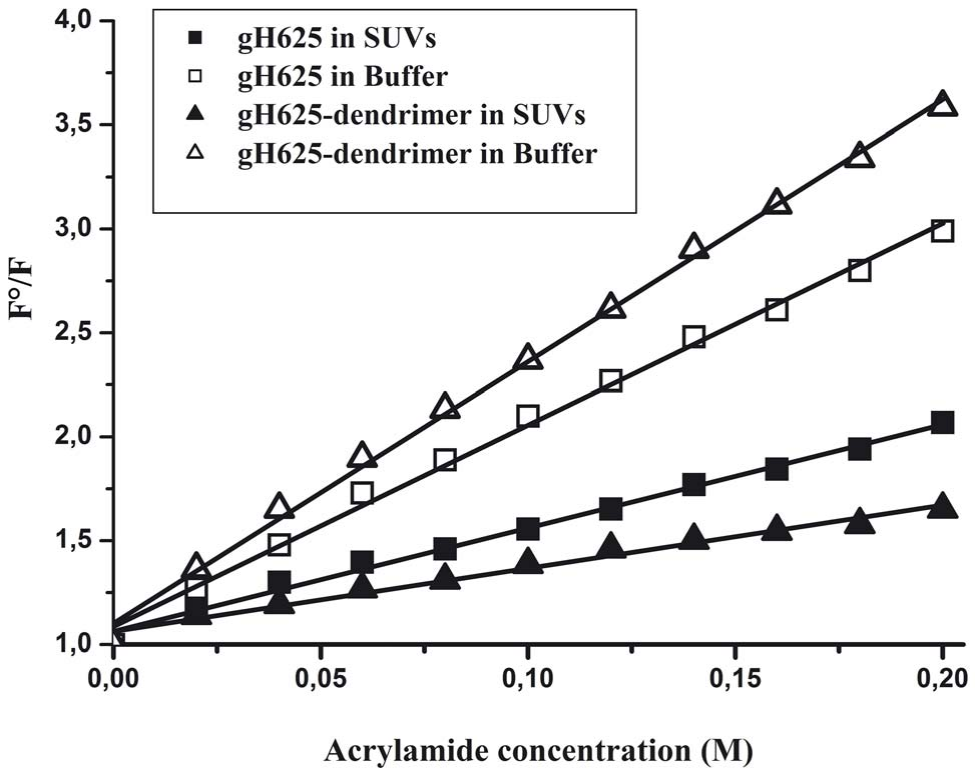
Stern-Volmer plots of acrylamide quenching of gH625 and gH625-Dendrimer in buffer (open symbols) and in LUVs (closed symbols).

The changes in the tryptophan fluorescence spectra upon binding of gH625 and gH625-dendrimer to lipid vesicles indicate their insertion into the hydrophobic bilayer. We further determined the accessibility of the tryptophan residue to acrylamide, a neutral, water-soluble, highly efficient quenching molecule that is unable to penetrate into the hydrophobic core of the lipid bilayer. The more deeply a tryptophan residue is buried, the less strongly it is quenched by acrylamide. Stern-Volmer plots for the quenching of tryptophan by acrylamide in buffer and in PC/Chol LUVs are shown in Figure 4.

Fluorescence of tryptophan decreased in a concentration dependent manner by the addition of acrylamide to the peptide solution both in the absence and presence of liposomes, without other effects on the spectra. However, in the presence of liposomes, less decrement in fluorescence intensity was evident, thus revealing that tryptophan is less accessible to the quencher in the presence of LUVs. K_sv_ values are reported in Table 2.

**Table 2:** Stern-Volmer (K_sv_) quenching constant calculated from the equation F^o^/F = 1+K_sv_[Q] (Q, quencher) for gH625 and gH625-dendrimer.

	K_sv_(M^−1^)
gH625 in Buffer	9.70±0.21
gH625 in LUVs	4.98±0.14
gH625-dendrimer in Buffer	12.60±0.22
gH625-dendrimer in SUVs	3.04±0.14

Analysis of the obtained values confirm that the gH625-dendrimer tryptophans are more exposed than those of gH625 in buffer; in fact the K_sv_ of gH625-dendrimer is higher. On the contrary, the K_sv_ value in LUVs of gH625-dendrimer is lower, suggesting that tryptophans are more buried in the bilayers, becoming more inaccessible for quenching by acrylamide. Comparison of the plots (Figure 4) and of the values of K_sv_ (Table 2) confirms that the tryptophans in gH625-dendrimer are more deeply inserted than in the isolated peptide.

### Membrane permeability studies

Increasing concentrations of gH625 and gH625-dendrimer were mixed with the same amount of PC/Chol SUVs pretreated with the fluorescent dye and valinomycin. We did not observe any enhanced recovery of fluorescence (quenched by the addition of valinomycin) with increasing amounts of the two molecules. Our results indicate that there is no electroporation effect due to the peptide or the peptide-dendrimer interactions with the SUVs.

### Tryptophan quenching experiments with Br-PC

**Figure 5:**
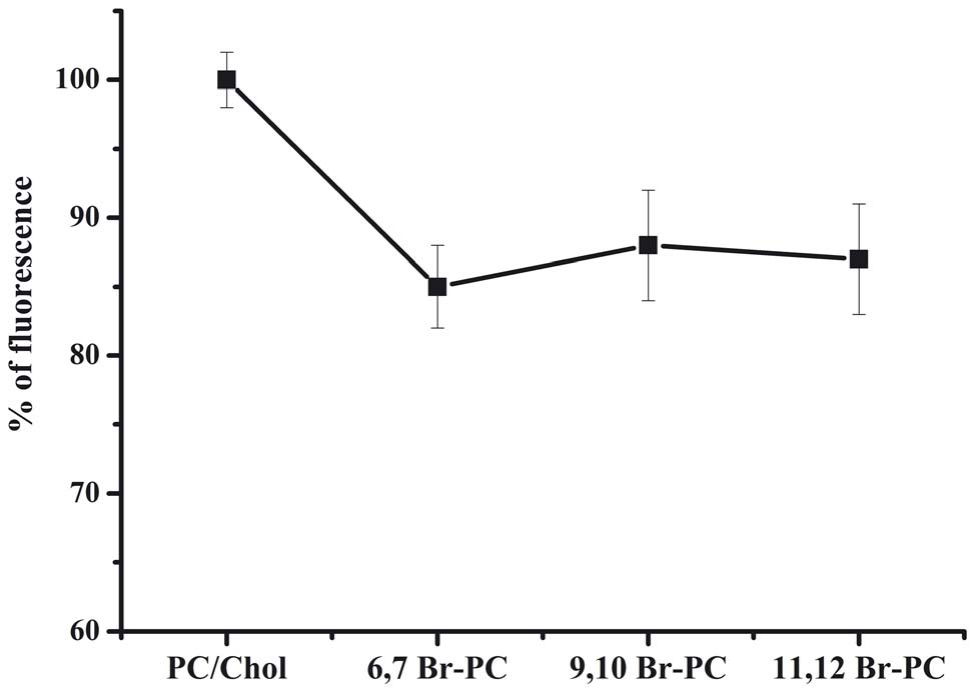
Percentage of tryptophan fluorescence for gH625−dendrimer in PC/Chol and in the presence of the probes 11,12-Br-PC, 9,10-Br-PC, and 6,7-Br-PC.

The position and the depth of the two molecules inside the bilayer can be investigated by measuring the relative quenching of the fluorescence of the Trp residue by the probes 11,12-Br-PC, 9,10-Br-PC, and 6,7-Br-PC, which differ in the position of the quencher moiety along the hydrocarbon chain. 6,7-Br-PC is a better quencher for molecules near or at the interface, while the other two are better probes for molecules buried deeply inside a membrane. We previously reported that the largest quenching of tryptophan fluorescence for gH625 was observed with 11,12- Br-PC vesicles and 9,10-Br-PC, while slightly less quenching was observed with 6,7-Br-PC [20]. These results indicate that, upon binding to vesicles, gH625 inserted into the membrane bilayer with the tryptophan side chain pointing toward the bilayer interior. We observed a different result for the gH625-dendrimer. There is a significant change compared to the control but the three spectra are superimposable, indicating that there is probably a distribution of the molecule inside the bilayer with the tryptophan localized at various depths (Figure 5).

### Binding Analysis by Surface Plasmon Resonance (SPR)

**Figure 6:**
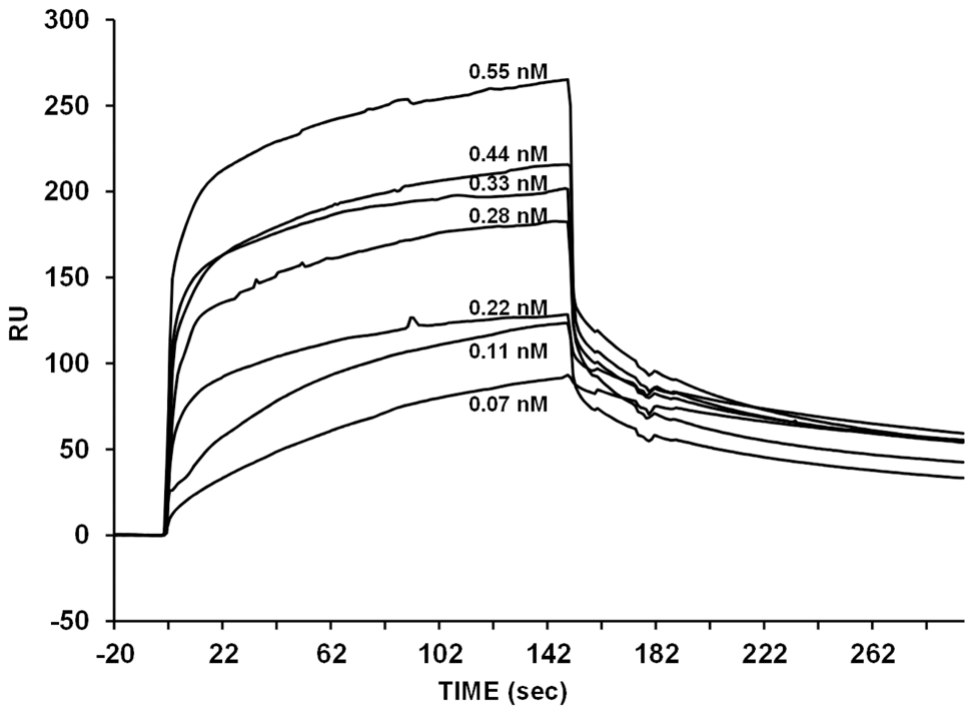
Sensorgrams of the binding between various concentrations of gH625-dendrimer with the HPA chip.

We utilized a BIAcore biosensor to investigate the mode of action of gH625-dendrimer and compared it with gH625 [18], [19]. PC/Chol monolayers were absorbed onto the HPA. Sensorgrams of the binding with monolayers are shown in Figure 6.

The sensorgrams revealed that the RU signal intensity increased as a function of the molecule concentration. This indicates that the amount of molecule bound to the lipids is proportional to its concentration. We employed numerical integration analysis that uses nonlinear analysis to fit an integrated rate equation directly to the sensorgrams [51]. When fitting the sensorgrams globally with the simplest 1∶1 Langmuir binding model, a poor fit was obtained (χ^2^>50), confirming that this model does not represent the lipid binding mechanism of the gH625-dendrimer. However, a significantly improved fit was obtained using numerical integration of the two-state reaction model of the binding sensorgrams, suggesting that, in analogy with the data previously obtained for the peptide alone [19], there are likely to be at least two steps involved in the interaction between the gH625-dendrimer and the hybrid bilayer membrane surface. In analogy with previous studies of peptide-membrane interactions using SPR [19], [50], [52], the first step may correspond to the actual binding to the surface, while the second step might be the insertion into the hydrophobic core of the membrane. A set of sensorgrams with different gH625-dendrimer concentrations was used to estimate the kinetic parameters. The average values for the rate constants obtained from the two-state model analysis are listed in Table 3 along with the affinity constant values (K_A_) and compared with the data previously obtained for the peptide gH625 [18].

**Table 3:** Association (k_a1_, k_a2_) and dissociation (k_d1_, k_d2_) rate constants obtained for the HPA chip using the two state model.

	gH625	gH625-dendrimer
**k_a1_**	(5.11±0.03)10^1^	(6.57±0.02)10^3^
**k_d1_**	(2.51±0.05)10^−2^	(6.95±0.03)10^−2^
**K_1_**	2.0 10^3^	9.4 10^4^
**k_a2_**	(5.22±0.05)10^−3^	(1.61±0.05)10^−2^
**k_d2_**	(110±0.06) 10^−4^	(4.93±0.09)10^−3^
**K_2_**	47	3.2
**K_A_**	1.00 10^5^	4.03 10^5^

The affinity constants K_1_ and K_2_ are for the first (K_1_ = k_a1_/k_d1_) and for the second (K_2_ = k_a2_/k_d2_) steps respectively, and the affinity constant (K_A_) determined as (k_a1_/k_d1_) × (k_a2_/k_d2_) is for the complete binding process. Standard deviations are reported in brackets.

The data indicate the main influence on the overall binding constant of the fast association rate and slow dissociation rate of the first step. If this step corresponds to the electrostatic interaction, these results suggest that electrostatic forces play an important role in the binding of membrane-active peptides.

It is interesting to note that gH625 and gH625-dendrimer have high K_1_, indicative of the first step corresponding to the electrostatic interaction is important for the binding and the higher value for gH625-dendrimer is probably influenced by the presence of several peptide molecules on the same dendrimer. The overall binding to the membrane bilayer is thus stronger for the gH625-dendrimer.

### Isothermal calorimetry

**Figure 7:**
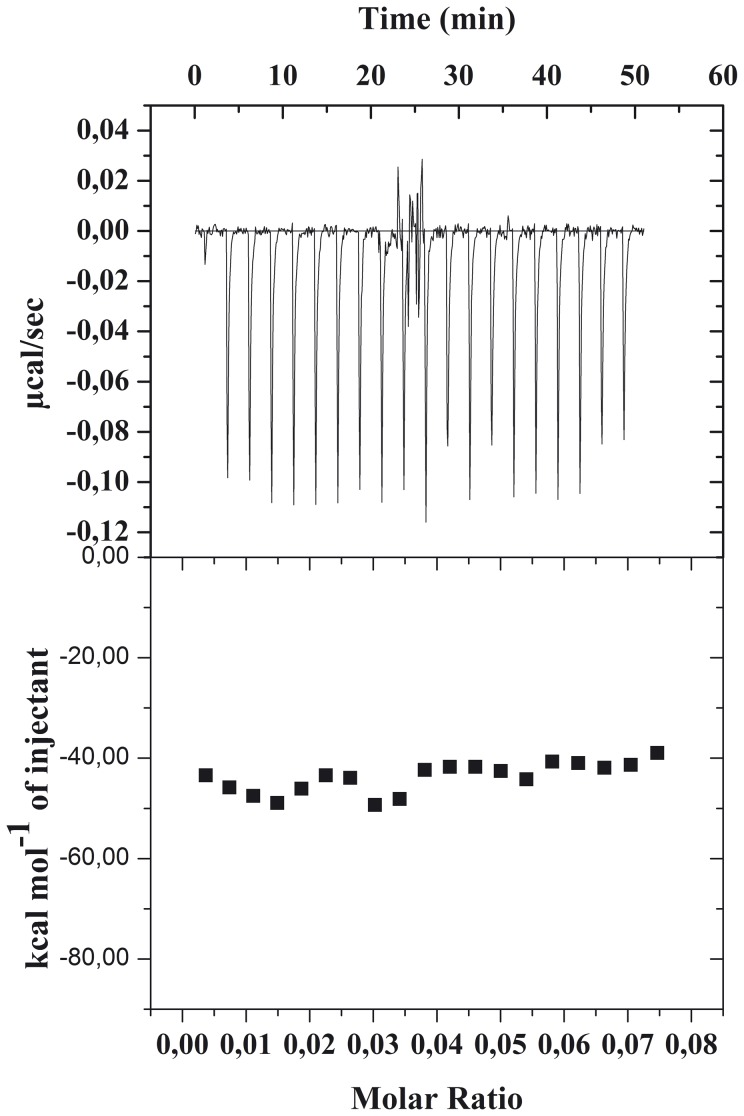
Isothermal titration calorimetry of SUVs with gH625-dendrimer at 25°C. The upper panel shows the calorimeter trace; the lower panel shows the molar enthalpy as evaluated by integration of the calorimeter traces.

Isothermal calorimetry can be applied to study membrane interactions in two different modes. In the first, the calorimeter cell contains lipid vesicles and the ligand is injected into the calorimeter cell, while in the second, the two solutions are exchanged. The two experiments provide different thermodynamic parameters. The first type of experiment allows to measure the reaction enthalpy ΔH (Figure 7).

In the second experiment, after each addition of lipid, the ligand is bound to the lipid vesicles and removed from bulk solution; as a consequence, with increasing lipid concentration in the reaction vessel, less and less ligand is available for binding and the heat of reaction is no longer constant but decreases with each lipid injection. The ΔH values determined in the two experiments should be identical, but the additional advantage of the second type of experiment is the ability to measure the binding isotherm and to evaluate the binding constant K.

The enthalpy change upon binding of the gH625-dendrimer to lipid vesicles was measured from the first type of experiment by injecting small aliquots of a gH625-dendrimer solution into concentrated SUVs. Under these conditions, the lipid is in large excess over the ligand during the whole experiment and the injected ligand is completely bound to the membrane surface if the binding constant is sufficiently large. Each injection experiment thus produces the same heat of reaction, Δh. Figure 7 shows the result of the injection of 2 µl aliquots of the 0.017 mM gH625-dendrimer solution (buffer: Hepes 5 mM, NaCl 100 mM, pH 7.4) into 0.033 mM PC:Chol (55∶45) SUVs at 25°C. The average heat of reaction observed after subtraction of the heat of dilution (measured in control experiments of peptidodendrimer into buffer injection) was −1.5 µcal. The analysis of the heat of reactions shows that the reaction is exothermic. Dividing the heat of reaction by the amount of injected molecule yields the molar enthalpy of binding, ΔH (−47.5 Kcal/mol) (Figure 7).

**Figure 8:**
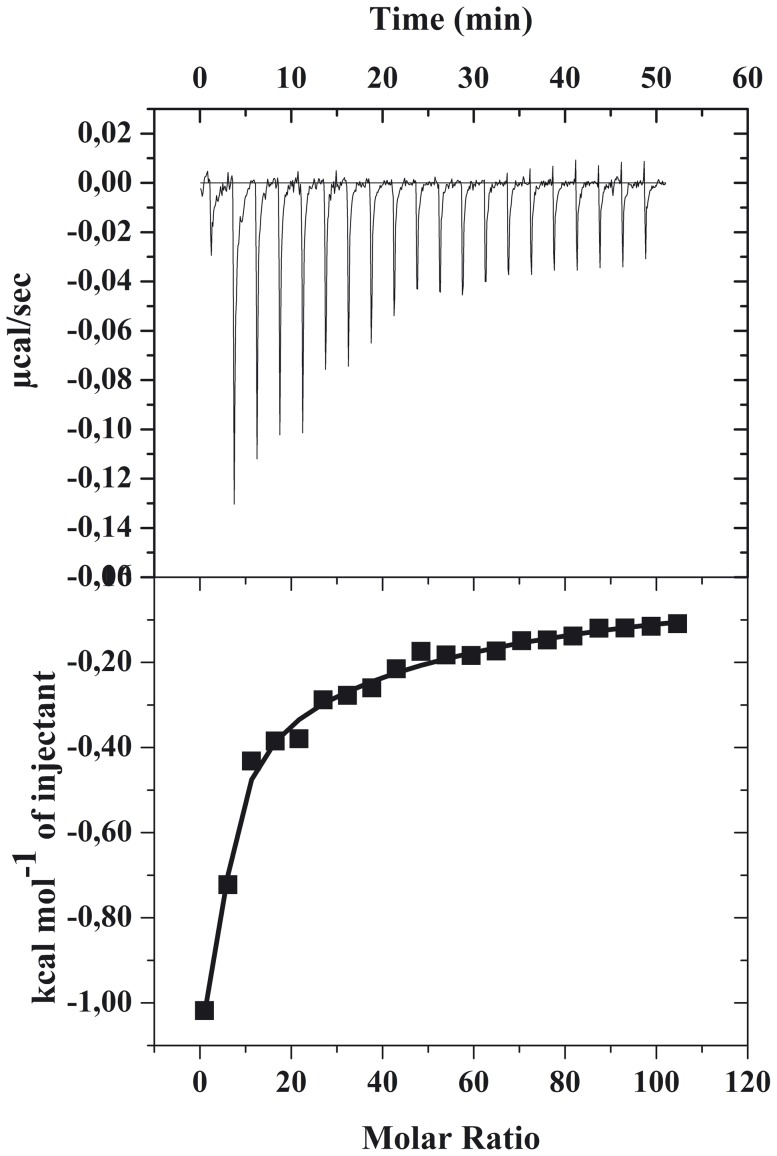
Isothermal titration calorimetry of gH625-dendrimer with SUVs at 25°C. The upper panel shows the calorimeter trace; the lower panel shows the molar enthalpy as evaluated by integration of the calorimeter traces.

In the second experiment, the gH625-dendrimer was contained in the calorimeter cell and lipid vesicles were injected. This allowed the determination of the binding isotherm. The molecule was employed at a concentration of 2.8 µM and each titration peak corresponds to a 2 µl injection of a 2 mM SUVs (20 injections) except the first one that corresponds to a 0.4 µl injection. The amount of heat released decreases with increasing injection number as less and less gH625-dendrimer remains free in solution. The heat of reaction values are shown in Figure 8 after subtraction of the heats of dilution obtained in control experiments of vesicle-into-buffer titrations.

If we denote with Δh the experimentally measured heat of reaction at the *k*th injection, then Σ^k^ *_1_*Δh*_k_* is the *cumulative* heat of the first *k* injections and Σ^n^ *_1_*Δh_k_ is the *total* heat of injection provided Δh_k_, the last injection step, is zero. It is possible to calculate the molar heat of reaction, ΔH, according to: 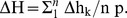
where *n* is the molar amount of the molecule in the calorimeter cell after *n* injections.

The ΔH values determined in the two experiments should be identical. However, the additional advantage of the second type of titration experiment is the possibility to measure the binding isotherm and to evaluate the binding constant *K*. The binding isotherm reports the dependence of the molar ratio of bound molecule per total lipid, X_b_, on the free molecule concentration in solution, C_f_, and can be derived using established procedures [53]. As for the fluorescence data, X_b_^*^ was calculated on the basis of the lipid present in the outer leaflet of the bilayer only since the molecule cannot cross the bilayer under the present experimental conditions.

The two essential parameters which constitute the binding isotherm, namely the degree of binding X_b_^*^ and the corresponding free molecule concentration C_f_ can thus be determined in a single titration experiment.

**Figure 9:**
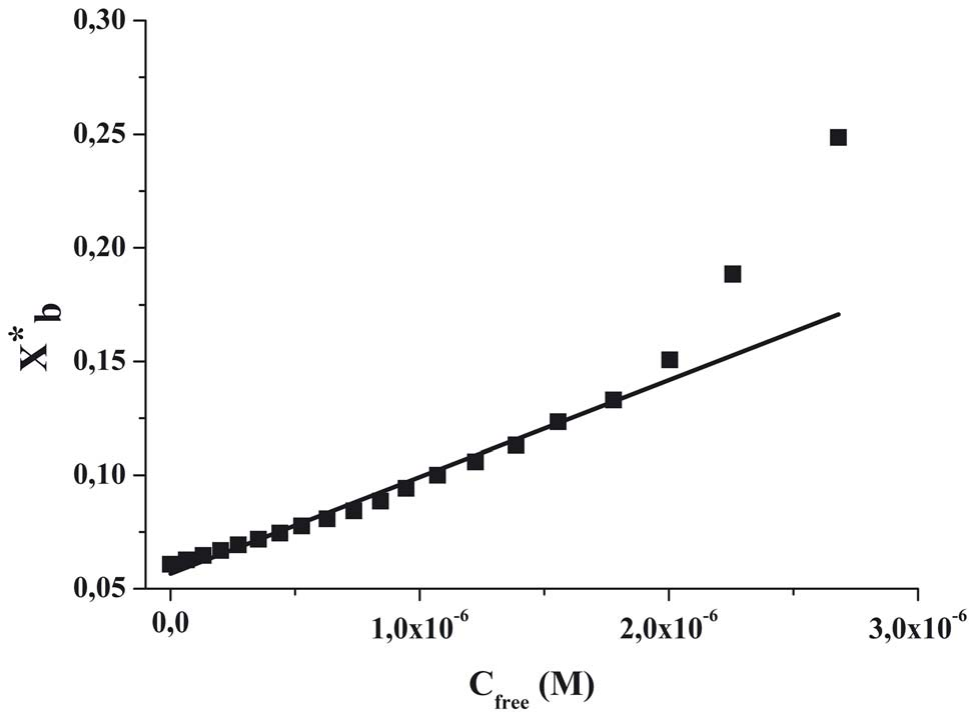
Binding isotherms for binding of gH625-dendrimer to SUVs at 25°C. Binding isotherms were derived from lipid into peptidodendrimer titrations as described in the text. The degree of binding is plotted against the free peptidodendrimer concentration. The solid line corresponds to the theoretical binding isotherm.

Hence the peptidodendrimer lipid-binding equilibrium is X_b_^*^ = f(C_f_), where f(C_f_) describes the functional dependence of X_b_^*^ on C_f_. The exact form of the functional dependence depends on the nature of the problem and provides information on the organization of the molecule within the membrane as was previously discussed for fluorescence data. A linear relationship is the exception rather than the rule; a straight line indicates a simple adhesion process. The shape of the binding isotherm was not linear indicating that the gH625-dendrimer accumulation at the surface is a complex phenomenon. In particular, also from isothermal calorimetry we obtained a binding isotherm characterized by a flattening at the origin, followed by steep rise, which could be interpreted as reflecting a process whereby peptides first incorporate into the membrane and then aggregate within. In our isotherms, the total extent of incorporation (X_b_^*^) slowly increases until a critical concentration is reached, where massive internal aggregation apparently starts to develop (Figure 9).

The surface partition coefficient K_p_ was estimated by extrapolating the initial slopes of the curves to C_f_ values of zero. The values of K_p_ (4.3 10^4^) obtained are within the range of those obtained for membrane-permeating bioactive peptides and its high value indicates that the tryptophans of gH625-dendrimer are embedded in the bilayer. Knowledge of the partition constant allows the calculation of the free energy of binding according to ΔG  =  -RTln(55.5 K), yielding ΔG = -8.6Kcal/mol (Table 4). The factor 55.5 corrects the cratic contribution since the concentration of the macromolecule in solution is measured in moles per liter and that in the membrane phase in moles of macromolecule per mole of lipid [54]. The relation ΔG = ΔH-TΔS, allows the calculation of ΔS  = −130 cal/mol K. Considering the complex interaction of the gH625-dendrimer with the bilayer, we may consider that this constant is model independent and describes the overall behavior. We obtained a considerably favorable enthalpic contribution and a unfavorable entropic contribution. This unfavorable entropy change may be attributed to the conformational change of gH625 from an unordered structure in aqueous solution to an ordered α-helix upon interaction with the membrane as previously shown by circular dichroism data in membrane mimetic environment [22] and as reported in literature for other peptides [55], [56]. Nevertheless, the overall free energy change is still large.

**Table 4:** Partition coefficient for the binding of gH625-dendrimer with PC/Chol obtained by isothermal calorimetry.

Method used for calculations	ΔH (Kcal/mol)	K	ΔG (Kcal/mol)	TΔS (Kcal/mol)	ΔS (cal/mol K)
Isothermal binding curve	−47.5±0.9[a]	4.3 10^4^	−8.6	−38.7	−130
One site Binding	−48±3	1.0 10^4^	−5.4	−42.6	−143
Sequential site binding	−6.3±0.5	7.6 10^4^	−6.6	+0.3	1
	−47.3±0.3	2.9 10^3^	−4.7	−42.6	−143

[a] This value is the one obtained from the titration of the lipid with the macromolecule.

In analogy with the SPR analysis, we decided to interpolate the experimental data reported in Figure 7 with two different models: the one site binding and the sequential binding site. From the one binding site we obtained a high χ^2^ and thus thermodynamic parameters similar to those obtained from the isothermal binding curve but with a high standard deviation. When we used the sequential binding (for 2 sites) we obtained lower χ^2^. The thermodynamic parameters are indicative of an initial interaction with the bilayer with a negative enthalpic change (−6.3 Kcal/mol) which is in the typical range of membrane interactions (from −2 to −10) [53] and a small favorable entropic component (1 Kcal/mol) which may be attributed to the partition from water into a nonpolar environment due to the release of the hydration shell. The second step may correspond to the insertion and the conformational change inside the bilayer. The enthalpic contribution is very favorable while the entropic contribution is negative. These results are similar to those obtained for membrane interacting peptides and has been previously attributed to the formation of an α -helix in the membrane bilayer [57]. The high binding constant for this step may allow us to conclude that membrane-facilitated helix formation is a strong driving force for the gH625-dendrimer binding to the lipid membrane.

## Conclusions

Dendrimers are well-defined highly branched structures that have attracted much interest in the biomedical field [58], [59], [60], [61]. Among their potential biomedical applications are drug delivery across the cell membrane, a complex structure composed of lipids and embedded proteins. In order to understand dendrimer's mechanism of action and to increase their targeting efficiency, the interaction with lipid bilayers needs to be unravelled. Model membranes, like liposomes, represent outstanding models due to their simple composition, ease of preparation and good stability.

Many reports are dedicated to the study of interactions between dendrimers and biological membranes. The type and strength of the interaction is dependent on charge and size of the molecule. Evidences suggest that dendrimers either create holes in a bilayer [29], [30], [31], [32], [33], [39], [62], [63], [64] or can be incorporated into lipid structure [35], [37], [45], [65]. Higher generation dendrimers cause greater disturbances in a lipid bilayer and positively charged dendrimers interact more effectively with liposomes [28], [35], [42], [43], [44], [45]. It has been proposed that at low concentrations, dendrimers can traverse the bilayer, whereas at higher concentrations, dendrimer: lipid micelles are created [63], [66].

The toxicity of PAMAM dendrimers generally increases with increasing generation number, which has been attributed to the increased number of positive charges per molar concentration and thus increased contact area of the dendrimer with the cell membrane [29]. The electrostatic interactions of cationic dendrimers with cell membranes probably induces nano-scale hole formation and membrane destabilization mechanisms [29], [30], [67]. It is thus necessary to remove positive charges, through the functionalization of external groups, from the surface of the dendrimer [68] to achieve better cytotoxicity profiles. In this contribution, we present a dendrimer with the external termini modified by a peptide, which has been proved to enhance cell membrane crossing [14], [69], [70].

The peptide gH625 has been previously characterized [16], [17], [18], indicating its strong interaction with LUVs without the formation of pores inside the bilayer, therefore able to reduce toxicity problems. We have previously shown that the peptidodendrimer is more effective than the native peptide at interacting and fusing with lipid membranes, showing its efficacy as a membrane-perturbing agent [22]. We proposed that the peptidodendrimer translocates across the bilayer without involving essentially the endocytic mechanism.

The mechanism of internalization has direct implications for the design of drug delivery, cell transfection and gene therapy agents. In order to gain a better understanding of this mechanism for the peptide functionalized dendrimer, it was essential to investigate possible disruption of cell membranes. Delivery agents that have the ability to pass through membranes without causing leakage are desirable as drug delivery vehicles.

All the results from the complementary techniques such as fluorescence spectroscopy, surface plasmon resonance and isothermal calorimetry, obtained in this contribution, indicate that the gH625-dendrimer has a high affinity for the membrane bilayer; moreover, we were able to determine via tryptophan quenching experiments with Br-PC that the molecule is deeply inserted inside the bilayer. We proved that the molecule is able to cause membrane fusion but not holes in the bilayer. The peptide coupled to the dendrimer is also able to assume a helical conformation when in membrane mimetic environment. The data obtained further support the hypothesis that the binding to the membrane bilayer can be divided at least into two steps. The first step is an adsorption process that leads to an increased macromolecule concentration on the membrane. The second step is a hydrophobic insertion which is characterized by penetration into the lipid bilayer and probably a conformational change of the peptide coupled to the dendrimer. The first step has a higher binding constant compared to the second step as obtained both from surface plasmon resonance and isothermal calorimetry. These data further support the view that the mechanism of lipid association plays a key role in the translocation activity and the hypothesis that gH625-dendrimer cellular uptake is associated with its ability to interact with membrane lipids and to temporarily affect membrane organization, thereby facilitating insertion into the membrane and translocation.

The translocating ability of our peptidodendrimer is different from the most commonly used cell penetrating peptides (such as the HIV derived TAT peptide) and from the mechanism hypothesized for PAMAM dendrimers, which do not translocate spontaneously across bilayers but rather are taken up by cells via endocytosis [21], [71].

Our peptido-dendrimer presents a very efficient membrane penetration ability and our findings could prove helpful for a better understanding of the advantages of combining dendrimers and peptides to design and develop new and effective drug delivery tools.
